# Jellyfish: integrative visualization of spatio-temporal tumor evolution and clonal dynamics

**DOI:** 10.1093/bioinformatics/btaf091

**Published:** 2025-02-25

**Authors:** Kari Lavikka, Altti Ilari Maarala, Jaana Oikkonen, Sampsa Hautaniemi

**Affiliations:** Research Program in Systems Oncology, Research Programs Unit, Faculty of Medicine, University of Helsinki, Helsinki 00014, Finland; Research Program in Systems Oncology, Research Programs Unit, Faculty of Medicine, University of Helsinki, Helsinki 00014, Finland; Research Program in Systems Oncology, Research Programs Unit, Faculty of Medicine, University of Helsinki, Helsinki 00014, Finland; Research Program in Systems Oncology, Research Programs Unit, Faculty of Medicine, University of Helsinki, Helsinki 00014, Finland

## Abstract

**Summary:**

Spatial and temporal intra-tumor heterogeneity drives tumor evolution and therapy resistance. Existing visualization tools often fail to capture both dimensions simultaneously. To address this, we developed Jellyfish, a tool that integrates phylogenetic and sample trees into a single plot, providing a holistic view of tumor evolution and capturing both spatial and temporal evolution. Available as a JavaScript library and R package, Jellyfish generates interactive visualizations from tumor phylogeny and clonal composition data. We demonstrate its ability to visualize complex subclonal dynamics using data from ovarian high-grade serous carcinoma.

**Availability and implementation:**

Jellyfish is freely available with MIT license at https://github.com/HautaniemiLab/jellyfish (JavaScript library) and https://github.com/HautaniemiLab/jellyfisher (R package).

## 1 Introduction

Intra-tumor heterogeneity plays a critical role in driving tumor evolution and therapy resistance (Dagogo-Jack and Shaw 2018). As a tumor progresses, proliferating cells accumulate unique genetic alterations, leading to the formation of genetically distinct subclones. These subclones can exhibit significant diversity across different regions of the tumor (spatial heterogeneity) and evolve over time (temporal heterogeneity), especially under the selective pressures of treatment. Accordingly, a bulk tumor sample contains a mixture of subclones, and the composition can vary significantly between different samples, reflecting the tumor’s dynamic complexity.

Heterogeneity in tumors is particularly noticeable in aggressive cancers, such as ovarian high-grade serous carcinoma (HGSC), where remarkable differences in subclonal composition have been observed between primary tumors and metastases, as well as across different stages of treatment (Bashashati *et al.* 2013, Lahtinen *et al.* 2023). Understanding both the spatial distribution and temporal evolution of subclones is essential for uncovering the mechanisms behind therapy resistance and disease progression.

Visualizing both the spatial and temporal dimensions of tumor evolution in solid tumors, although vital, presents a significant challenge. Current tools, such as fishplot (Miller *et al.* 2016) and clevRvis (Sandmann *et al.* 2022), were primarily designed for non-solid tumors, which lack a spatial component, thus focusing solely on the temporal aspects of tumor evolution and lacking the ability to capture spatial heterogeneity. Tools like evoFreq (Gatenbee *et al.* 2019) and TimeScape (Smith *et al.* 2017), while similar in design to fishplot, share these same limitations. Conversely, MapScape (Smith *et al.* 2017) enables detailed visualization of spatial heterogeneity, anchoring samples to anatomical locations, but it cannot integrate temporal dynamics within the same plot. ClonEvol (Dang *et al.* 2017), which also infers clonal ordering, provides separate phylogenetic trees and bell plots, but requires users to integrate and interpret the results.

**Figure 1.**
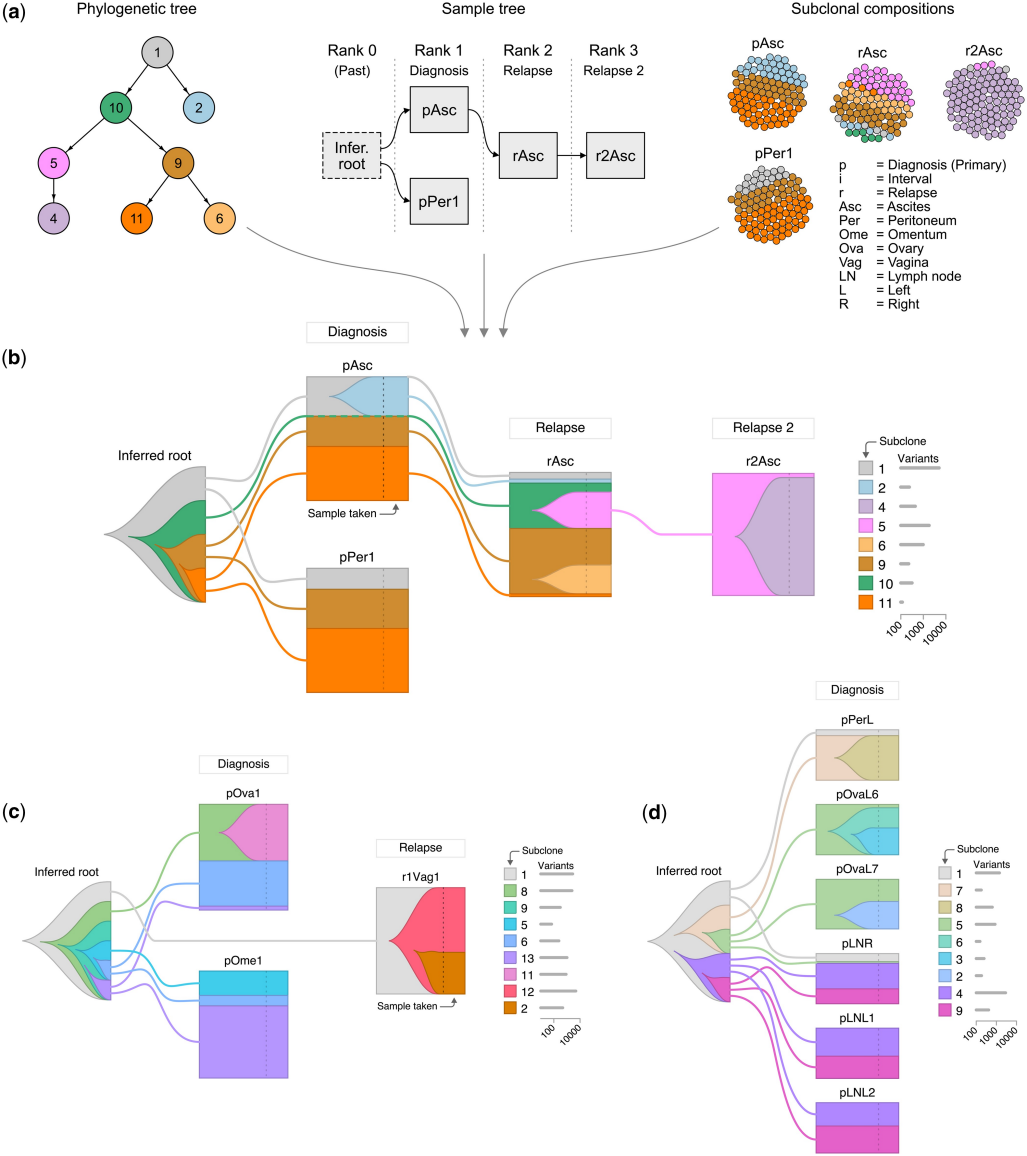
Overview and examples of Jellyfish. (a) The input data frames for Jellyfish. The phylogenetic tree (left) and subclonal compositions (right) can be estimated using ClonEvol or other similar tools. The user provides the sample tree (middle), which represents the assumed metastasis route of the tumor. Samples without a specified parent are automatically assigned as children of the inferred root sample. (b) The resulting Jellyfish plot of patient EOC677. The columns (ranks) in this plot correspond to treatment stages when the samples were acquired. For instance, *Diagnosis* represents tissue samples collected during primary debulking surgery, while *Relapse 2* represents a later time point within the relapse stage. This plot uses the same ClonEvol color scheme we used in (Lahtinen et al., 2023). (c) Jellyfish of patient EOC69 shows a new, trunk-derived subclones in relapse. (d) Jellyfish of patient EOC495 demonstrates the spatial dimension with samples ordered by their similarity. In addition, the phylogeny-aware color scheme emphasizes subclone four, which harbors numerous unique variants.

To bridge the gap in visualizing temporal and spatial tumor evolution, we developed Jellyfish, a novel visualization tool that simultaneously represents both spatial and temporal dimensions of tumor evolution in a single, unified plot. The preliminary version of the Jellyfish visualization design was introduced in our earlier work (Lahtinen *et al.* 2023), in which the plots were drawn manually in a vector-art editor, and thus, their scalability and usability were severely limited. Here, we present a fully automated implementation of Jellyfish, available as both a JavaScript library and application, as well as an R package called *Jellyfisher*, which allows users to generate Jellyfish plots from data frames representing tumor phylogeny, clonal compositions, and assumed metastasis routes of the samples (Fig. 1a). Additionally, Jellyfisher can directly read necessary data from ClonEvol output for instant visualization.

## 2 Implementation

The core concept of Jellyfish is to integrate two trees into a single plot: a *phylogenetic tree*, representing the evolutionary relationships of subclones, and a *sample tree*, which reflects the assumed metastasis route of the tumor. Samples within the sample tree are organized into *ranks*, which either represent the sample’s depth in the tree or, for example, correspond to treatment stages, time points, or the assumed timing of a metastasis event. Paths in the sample tree can skip ranks when necessary, with the only restriction being that a sample’s parent must be placed in an earlier rank. This way, ranks enable flexibility in representing the temporal evolution of the tumor in different scenarios (see Supplementary Notes) and allow for capturing the tumor’s spatial heterogeneity at different time points.

To show the subclonal evolution, Jellyfish embeds the phylogenetic tree into the sample tree, making it easy to perceive where each subclone first emerges and how it is inherited by other samples or passed on through its descendants (Fig. 1b).

### 2.1 The key visual elements of Jellyfish

A Jellyfish plot consists of two types of elements: *samples* and *tentacles*. Each sample is represented as a rectangle, where subclones are stacked vertically to show the sample’s subclonal composition. The samples are arranged into columns based on their ranks, which can optionally include titles indicating, e.g. the treatment stage when the samples were acquired. When a subclone first emerges in the sample tree, Jellyfish depicts it using a *bell* shape resembling a logistic growth curve. These emerging bells form a nested structure reflecting the phylogenetic relationships of the subclones.

Tentacle bundles connect the samples, with each tentacle representing a subclone that exists in both the parent and child samples. These bundles illustrate the structure of the sample tree and help trace the inheritance of subclones between samples.

### 2.2 Placement of emerging subclones

The emergence of subclones is trivial in a linear sample tree. However, as most trees are branched, Jellyfish places the emerging subclones by finding the lowest common ancestor (LCA) for each clade, i.e. the founding subclone and all its descendants, in the sample tree. These founding subclones are then visualized as bells in the sample rectangles. However, if the proportion of the subclone in the LCA sample is zero, the subclone must emerge in an earlier, possibly inferred sample.

### 2.3 Inferred samples

Sometimes samples lack a parent. For instance, if multiple samples have been acquired simultaneously in the initial diagnostic biopsy, they do not have a common parent in an earlier time point. However, to identify the LCA for a subclone present in these concurrent samples, Jellyfish infers a common parent sample. This inferred sample represents a hypothetical sample acquired at an earlier time point, where the subclone first emerged. Jellyfish visualizes the inferred sample as a bell shape, forming the “head” of the jellyfish. Since the subclonal proportions in this inferred sample are unknown, all subclones are displayed in equal proportions.

### 2.4 Meaningful order for samples within ranks

To aid the interpretation of spatial heterogeneity, Jellyfish arranges the samples within each rank based on the similarity of their subclonal compositions, clustering similar samples together. Jellyfish uses two complementary methods to determine the optimal order: the *phylogenetic center of mass* and *Jensen–Shannon (JS) divergence*.

For the phylogenetic center of mass, Jellyfish assigns an index number to each subclone by traversing the phylogenetic tree in depth-first order. It then calculates a weighted sum for each sample, where the weights correspond to subclone frequencies. This value represents the sample’s average position in the phylogenetic tree, which can be used to order the samples.

However, when samples are polyphyletic, i.e. they contain subclones from two or more highly divergent phylogenetic branches, the center of mass may not always produce an adequate clustering. To address this, Jellyfish also computes the JS divergence between the subclonal compositions of each pair of samples. These two measures are included in an objective function to find the optimal arrangement of samples within each rank (see Supplementary Notes).

### 2.5 Phylogeny-aware color scheme

Inspired by the work of Krzywinski (2016), Jellyfish generates a phylogeny-aware color scheme for subclones. By using the OKLCH color space, derived from the Oklab perceptual color space (https://bottosson.github.io/posts/oklab/), hue, chroma, and lightness can be adjusted independently without visual artifacts. Jellyfish first assigns a unique hue to each subclone based on its depth-first position in the phylogenetic tree, dividing the color wheel into equal segments. Next, chroma and lightness are assigned according to the total phylogenetic branch length, typically representing the number of mutations accumulated in the subclone. This approach results in subclones with more mutations appearing darker and more vibrant, drawing attention to those that are highly evolved in the visualization (Fig. 1c and d).

As Krzywinski noted, mechanistic adjustments of lightness can produce unpleasant, murky shades like dark yellow, which appears as brown. To prevent this, Jellyfish blacklists problematic colors and rotates the color wheel to find visually appealing combinations, ensuring an attractive and harmonious color scheme.

### 2.6 Interactivity

While simple Jellyfish with few samples and subclones are easy to interpret from a static plot, more complex cases benefit from interactivity. Jellyfish generates interactive plots that allow users to highlight subclones or clades by clicking or hovering over them. Hovering also displays additional details, such as clonal prevalences (proportions) and the number of unique mutations in each subclone. Moreover, although we optimized the default layout parameters carefully to provide a visually appealing and informative plot, users can interactively adjust the parameters to customize the appearance of the Jellyfish plot.

## 3 Results

We demonstrate the utility of Jellyfish using a dataset from our previous study on HGSC (Lahtinen *et al.* 2023), which included two cohorts: a discovery cohort of 55 patients (manually drawn jellyfish plots) and a validation cohort of 93 patients (no plots drawn). Here, we present automatically generated Jellyfish plots for both cohorts, which are available as example data, explorable at https://hautaniemilab.github.io/jellyfish/, and discuss three select plots in more detail.

The phylogenies and subclonal compositions were estimated using ClonEvol, as described in Lahtinen *et al.* (2023). For the sample trees, we used the following method: for each sample, we checked whether an earlier time point included exactly one sample from the same anatomical location. If such a sample existed, it was assigned as the parent; otherwise, the inferred root sample was used as the parent.

Patient EOC677 (Fig. 1b) shows temporal evolution in the ascites samples in response to multiple lines of therapy given between the time points, culminating in subclone 4 outcompeting earlier subclones. Interestingly, subclone 10 avoided detection in the diagnosis samples (pAsc and pPer1), although it is an ancestor of subclones 9 and 11.

In patient EOC69 (Fig. 1c), a clade originating from the founding subclone 8 predominates the diagnosis samples (pOva1 and pOme1). However, this clade disappears during treatment, and a new clade (subclone 12) derived from the trunk (subclone 1) emerges in the relapse sample (r1Vag1). This new clade shows significant evolutionary divergence, with a branch length (number of mutations) that surpasses both the trunk and subclone 8.

In patient EOC495 (Fig. 1d), the lymph nodes (pLNR, pLN1, and pLN2) primarily contain the clade originating from the founding subclone 4. Interestingly, the right lymph node (pLNR) contains a trace amount of subclone 5, the founding subclone of the clade predominating in the ovary samples (pOvaL6 and pOvaL7), suggesting polyclonal seeding.

## 4 Conclusions

With more clinical trials collecting samples from various anatomical regions over multiple time points, such as TRACERx (Jamal-Hanjani *et al.* 2014) and DECIDER (Lahtinen *et al.* 2023), advanced tools for visualizing spatio-temporal tumor evolution are urgently needed. To address this, we developed Jellyfish, software that integrates phylogeny, subclonal compositions, and both spatial and temporal dimensions into a single plot. Jellyfish’s flexibility allows it to be applied to a wide range of scenarios. For instance, the ranks can represent actual time points when samples were acquired or the assumed evolutionary time points, such as the emergence of metastases (see Supplementary Notes). Ultimately, Jellyfish facilitates straightforward interpretation of clonal dynamics, providing critical insights into tumor progression.

## Supplementary Material

btaf091_Supplementary_Data

## Data Availability

The Jellyfish JavaScript library and application are available on GitHub (https://github.com/HautaniemiLab/jellyfish) and deposited in Zenodo (https://doi.org/10.5281/zenodo.14697217). The Jellyfisher R package is available on GitHub (https://github.com/HautaniemiLab/jellyfisher) and deposited in Zenodo (https://doi.org/10.5281/zenodo.14698677). The phylogeny and clonal composition data used for the Jellyfish plots in this article are included as example data in both the Jellyfish and Jellyfisher packages, which are archived in Zenodo. All raw sequencing data are available at the European Genome-phenome Archive (EGA) under accession number EGAS00001006775.
