# Supplementary material for: Jellyfish: integrative visualization of spatio-temporal tumor evolution and clonal dynamics
Source: Bioinformatics. 2025 Feb 25;41(3):btaf091. doi: 10.1093/bioinformatics/btaf091 (PMC11897425; doi:10.1093/bioinformatics/btaf091)
Supplement: btaf091_Supplementary_Data [file btaf091_supplementary_data.pdf]

Jellyfish: Supplementary notes

Kari Lavikka Altti Ilari Maarala Jaana Oikkonen Sampsa Hautaniemi

1 Alternative sample trees

In the example dataset bundled with Jellyfish and Jellyfisher, the sample trees were constructed as follows: For each sample, we checked whether an earlier time point included exactly one sample from the same anatomical location. If such a sample existed, it was assigned as the parent; otherwise, the *inferred root* was used as the parent. However, this mechanistic approach may not always produce biologically credible sample trees.

1.1 Adjusting parents

Jellyfish offers considerable flexibility in defining the sample tree, enabling users to explore alternative configurations by adjusting the parent of each sample. For instance, while the sample tree depicted in the first panel of Figure S1 might seem implausible, a minor adjustment to the parent-child relationships can result in a more credible tree structure.

1.2 Adjusting ranks

In Jellyfish plots, samples are arranged into columns based on their ranks. Ranks serve as an abstraction that supports various use cases, such as representing different time points of the treatment or illustrating the depth of samples within the sample tree.

For example, in Patient EOC495 (Figure 1d in the main text), all samples are initially assigned to the diagnosis time point and connected to the inferred root. However, an alternative interpretation might suggest that the lymph node samples (pLNR, pLNL1, and pLNL2) represent later metastatic events rather than being contemporaneous with the primary tumor.

Jellyfish's flexibility allows users to modify the sample tree topology and optionally adjust ranks to suit their interpretation. In Figure S2, we illustrate an alternative tree where the lymph nodes are derived sequentially from each other rather than directly from the inferred root. Additionally, by adjusting the ranks in Figure S3, the first lymph node sample is moved further to the right, suggesting a progression over time and emphasizing the impression of later development.

2 Handling non-aberrant samples

In tumor evolution studies, the primary focus is typically on the subclonal compositions of tumor cells. Consequently, the root node in the phylogenetic tree generally represents the founding clone. However, datasets may also include non-aberrant cells, reflecting tumor purity below 100%.

To account for this scenario, a new root can be added to the phylogenetic tree to represent the normal, non-aberrant cells. Furthermore, Jellyfish offers options to customize the color and tentacles associated with this new "clone," enabling clear visual distinction from the aberrant subclones (Figure S4).

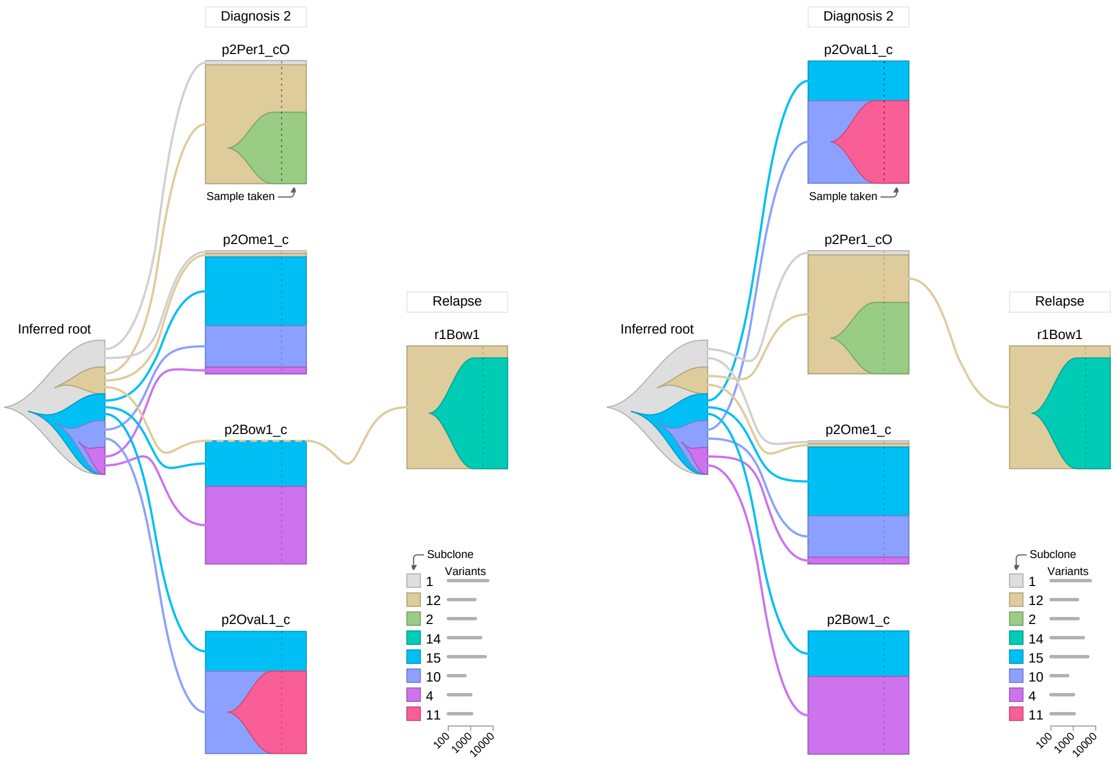

(a) Default sample tree

(b) Adjusted sample tree

Figure S1: In the example dataset for patient EOC809, the *r1Bow1* (bowel) sample was originally placed under the earlier bowel sample *p2Bow1_c*, which contains no traces of subclone 12. To address this implausibility, we moved *r1Bow1* under a new parent, *p2Per1_cO* (peritoneum), which has a higher prevalence of subclone 12 and is still a plausible source due to its anatomical proximity.

3 Layout optimization

Jellyfish organizes samples into vertically centered columns (ranks), creating a symmetric layout that minimizes empty space and enhances visual appeal. However, the order of samples within each column significantly impacts the interpretation and readability of the plots. Poor ordering can result in excessively long and tangled tentacle bundles, making the plot difficult to interpret. Additionally, placing samples in a random order within a column can obscure patterns of heterogeneity, hindering insights into sample relationships. Ideally, samples should be ordered by similarity while keeping tentacle bundles as short as possible, but these goals often conflict.

To address these challenges, Jellyfish employs a cost-based optimization procedure to determine the order of samples within each column. The cost function combines several factors, including the total length of tentacle bundles, deviations from the phylogenetic center of mass ordering (see main text), pairwise subclonal divergence between consecutive samples, and the alignment of tentacle bundles with the phylogenetic center of mass. Users can adjust the relative weights of these factors to influence the layout. Figure S5 illustrates the effect of modifying the `pathLength` parameter on the layout for patient EOC69.

Because finding a single combination of weights that works well for all datasets is challenging, Jellyfish provides carefully selected default settings optimized for most use cases, while still allowing users to customize parameters to suit specific datasets.

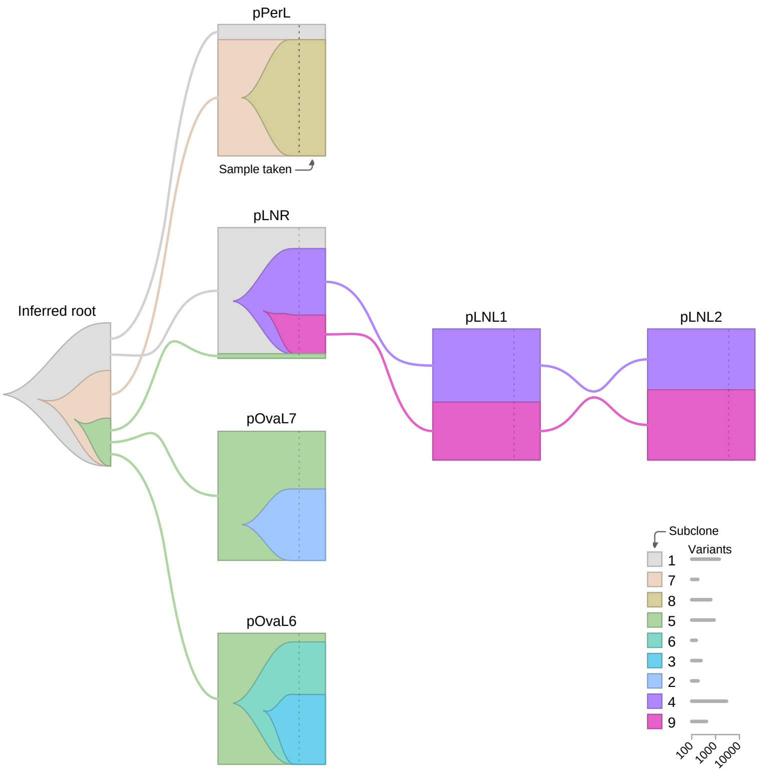

Figure S2: An alternative sample tree for patient EOC495, where the pLNR gives rise to the pLNL1 and pLNL2 samples. Here the ranks are used to show the samples' depths in the tree, without any time point information.

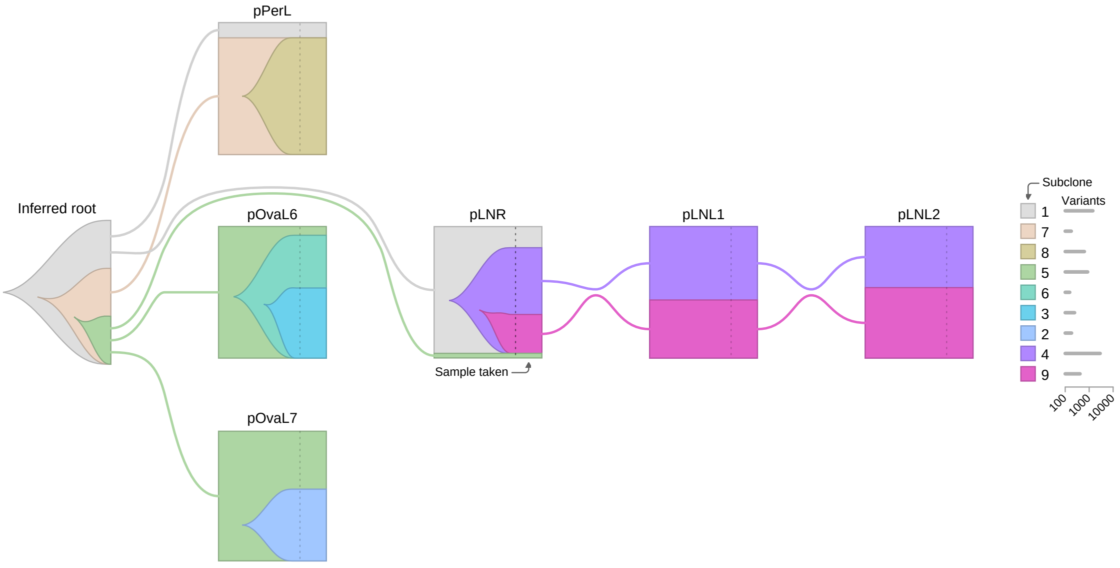

Figure S3: Another alternative sample tree for patient EOC495. If we assume that the lymph node samples are even later developments, we can manually assign them ranks that places them to the right of the diagnosis samples.

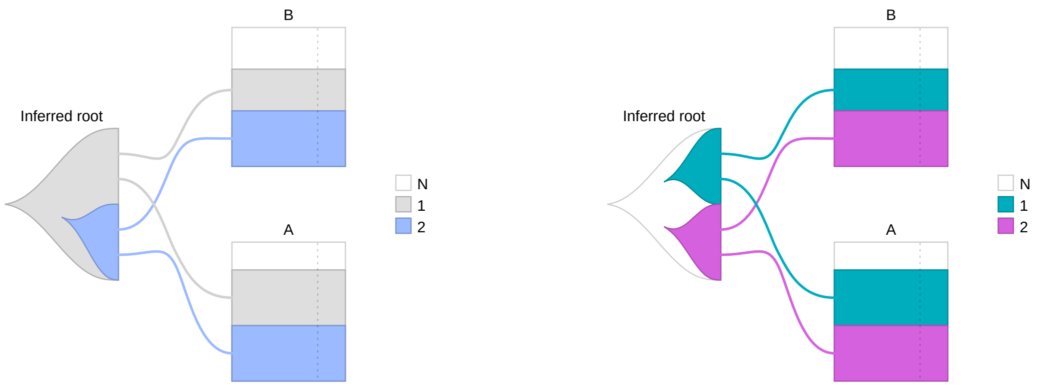

(a) Single clone

(b) Multiple independent clones

Figure S4: Showing non-aberrant samples in Jellyfish plots. In (a), there is a single founding clone (shown as gray as usually). The non-aberrant cells are shown as white and no tentacles are connected to them. In (b), there are two independent clones, and the non-aberrant cells are shown as the parent of the two clones in the *inferred root*.

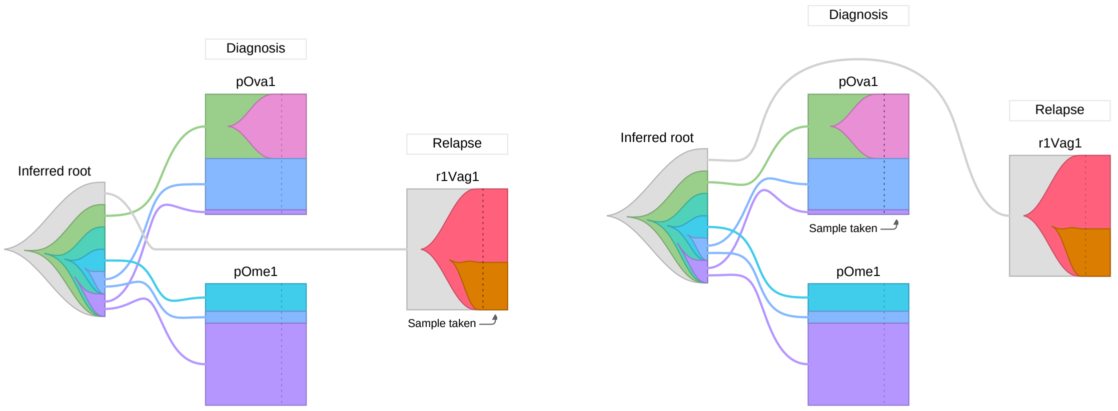

(a) Default layout

(b) Adjusted layout

Figure S5: Effect of layout optimization parameters on patient EOC69. (a) With default parameters, the layout prioritizes minimizing the path length between the *inferred root* and *r1Vag1*. (b) Slightly decreasing the `pathLength` parameter shifts the layout to prioritize the phylogenetic center of mass. This adjustment causes the gray tentacle to pass above *pOva1*, aligning with the subclone order visible in the *inferred root* sample.
